# Risk Factors for Acute Hemorrhagic Rectal Ulcers after Bypass Surgery for Chronic Limb-Threatening Ischemia

**DOI:** 10.3400/avd.oa.24-00125

**Published:** 2025-04-01

**Authors:** Yohei Kawai, Masayuki Sugimoto, Takuya Osawa, Changi Lee, Shuta Ikeda, Kiyoaki Niimi, Hiroshi Banno

**Affiliations:** Department of Surgery, Division of Vascular and Endovascular Surgery, Nagoya University Graduate School of Medicine, Nagoya, Aichi, Japan

**Keywords:** CLTI, bypass surgery, AHRU

## Abstract

**Objectives:** Acute hemorrhagic rectal ulcer (AHRU) occurs with a sudden onset of painless bloody stools and is caused by impaired blood flow in the rectal mucosa due to arteriosclerosis or prolonged bedridden status. Little information is available about AHRU in patients with chronic limb-threatening ischemia (CLTI). This study aimed to identify factors related to AHRU among CLTI patients after bypass surgery.

**Methods:** Between 2019 and 2023, we enrolled 80 CLTI patients at our institution who underwent bypass surgery using autogenous veins. Data were collected prospectively and supplemented with retrospective medical record reviews. Information regarding demographic and clinical characteristics was collected. The outcomes of patients without AHRU (non-AHRU group) and those with AHRU (AHRU group) were compared. Logistic regression analysis was used to assess factors associated with AHRU after bypass surgery.

**Results:** During the study period, 6 of the 80 patients (7.5%) experienced AHRU after bypass surgery. There was no significant difference in the global limb anatomic staging system (GLASS) or wound ischemia and foot infection (WIfI) stage between the 2 groups. The percentage of patients taking oral steroids was significantly greater in the AHRU group. In addition, the AHRU group had a significantly greater percentage of postoperative ambulatory failure and a longer hospital stay. In the univariate analysis of factors associated with the incidence of AHRU after bypass surgery, steroid use (odds ratio [OR], 13.8; 95% confidence interval [CI], 2.19–86.9; P = 0.005) and nonambulatory status after surgery (OR, 7.22; 95% CI, 1.26–41.4; P = 0.026) were significant factors.

**Conclusions:** Steroid use and postoperative nonambulatory status were associated with AHRU after bypass surgery for CLTI.

## Introduction

Acute hemorrhagic rectal ulcer (AHRU) is a condition that presents with painless bleeding from the rectum and often results in a critical outcome. It is more common in elderly people with underlying medical conditions and is considered to be associated with impaired activities of daily living (ADL). Although there have been several reports about AHRU, its risk factors have not been sufficiently investigated due to the small number of cases.^1–19)^

Chronic limb-threatening ischemia (CLTI) is associated with amputation, death, and deterioration in quality of life, and it is a condition requiring revascularization. Bypass surgery using an autologous vein is the standard treatment for CLTI, but it is more invasive than endovascular therapy.^20)^ Some patients who undergo bypass surgery may experience postoperative deterioration in ADL and suffer from AHRU during their hospital stay. However, few reports have shown an association between CLTI and AHRU. The aim of this study is to assess the risk factors for AHRU after bypass surgery for CLTI.

## Patients and Methods

### Study population

This clinical investigation analyzed the preoperative medical risk factors, surgical characteristics, and outcomes of 80 CLTI patients who underwent bypass surgery at our institution between 2019 and 2023.

Data were collected from a prospectively maintained database. Patient records were carefully reviewed retrospectively. This study was conducted in accordance with the Declaration of Helsinki. The Nagoya University School of Medicine Institutional Review Board approved the study (approval number: 2023-0265). All patients provided written informed consent prior to the intervention and data collection.

### Revascularization procedures and postoperative management

In our hospital, arterial revascularization is considered for all CLTI patients. Blood sampling data, cardiac status (electrocardiography, coronary angiography, and echocardiography), respiratory function, and other parameters were routinely assessed. The treatment strategy for each patient was discussed by the vascular surgical team, and the most appropriate treatment was selected on the basis of the patient's comorbidities, ambulatory status, venous status, wound condition, and anatomical features. Surgical bypass tends to be preferred as the first choice, particularly in patients with large tissue loss and lower leg lesions. The technical details of the vein graft bypass procedure have been published previously.^21,22)^ The distal anastomotic site was chosen based on the optimal runoff vessel to the ischemic wounds. Proximal anastomoses were performed with 6-0 polypropylene sutures, and distal anastomoses were performed with 7-0 polypropylene sutures. For distal anastomoses, we used the non-dissection method with pneumatic tourniquets to control blood flow. The greater saphenous vein (GSV) was the preferred conduit if it had a diameter greater than 2 mm, as determined by preoperative duplex scan imaging. If the GSV was unavailable, the lesser saphenous or an arm vein was used. When a single vein was too short, a spliced vein graft was created using 7-0 sutures. The policy is not to perform bypass surgery in patients with infections beyond the ankle joint, severe dementia, or gait failure prior to the onset of CLTI.^21,22)^

Patients taking oral steroids have preoperative steroid coverage, depending on the anesthesiologist's judgment, because their adrenal cortical function is suppressed.

Heparin was intravenously administered for 24 hours, and prostaglandin E1 was also infused for several days postoperatively. All patients subsequently received antiplatelet drugs. Recently, low-dose rivaroxaban has been administered in addition to aspirin in patients with acceptable renal function.

With respect to actual physiotherapy after bypass surgery, early mobilization from the day after surgery is encouraged, with close attention paid to vital signs. In patients with ischemic wounds, gait training is also given as early as possible, with thorough unloading.

### Definitions

**Figure 1:**
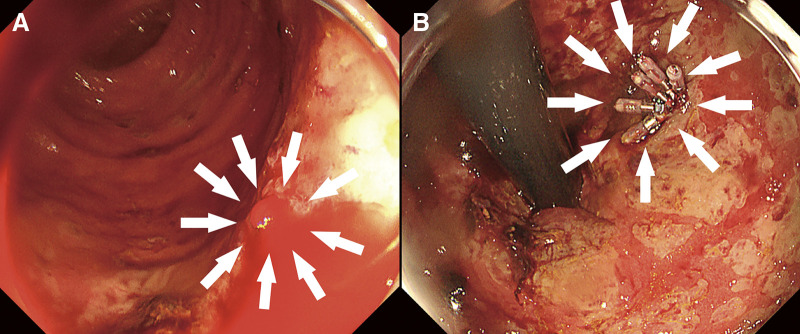
Fig. 1 Endoscopic findings. (**A**) Active bleeding was observed from the lower rectum (white arrows). (**B**) Endoscopic treatment was performed using clips (white arrows).

In this study, we included patients who underwent bypass surgery for CLTI. Coronary artery disease was defined as abnormal coronary angiography and previous myocardial infarction, open coronary artery surgery, or percutaneous coronary revascularization. Cerebrovascular disease was defined as patients who had suffered a stroke in the past or who had carotid artery lesions that had previously undergone carotid artery stenting or carotid endarterectomy. Hypertension, dyslipidemia, and diabetes mellitus were diagnosed in patients receiving relevant active treatment or dietary therapy. A positive smoking history was defined as any prior smoking. Ambulatory status was defined as the patient's functional status immediately before the initial operation. Patients were considered nonambulatory if they were wheelchair-bound or bedridden. AHRU was defined as an ulcerative lesion in the lower rectum within 5 cm of the dentate line, identified by endoscopy, with sudden, painless, fresh bloody stools (**Fig. 1**).

### Clinical endpoints

The goals of this study were to compare patients with and without AHRU after bypass surgery for CLTI and to analyze factors associated with AHRU.

### Statistical analysis

Normally distributed continuous variables are expressed as the means ± standard deviations. Medians and interquartile ranges are presented for other continuous variables. Categorical variables are presented as percentages. The JAPAN Critical Limb Ischemia Database (JCLIMB) prediction model was used to calculate the estimated 30-day mortality and major amputation rate, the estimated 2-year survival rate, and the amputation-free survival (AFS) rate.^23,24)^ Statistical significance was calculated and compared between the 2 groups using the χ^2^ test or unpaired t test, as appropriate. Logistic regression analysis was used to assess the associations between each variable and AHRU after bypass surgery. *P* values <0.05 were considered statistically significant. All statistical analyses were performed using IBM Statistics Statistical Package for Social Science (SPSS) version 29 (IBM Corp., Armonk, NY, USA).

## Results

During the study period, 80 CLTI patients underwent bypass surgery. According to the JCLIMB prediction model, the estimated 30-day death and major amputation rate, the estimated 2-year survival rate, and the AFS rate were 3.8% [2.3,6.2], 75% [62,84], and 67% [53,76], respectively. Seventy-four patients did not develop AHRU (92.5%; non-AHRU group), but 6 patients developed AHRU (7.5%; AHRU group). **Table 1** summarizes 6 patients with AHRU.

**Table:** Table 1 Patients with AHRU

Case	1	2	3	4	5	6
Days to the onset of AHRU after surgery	7	10	15	26	65	7
Age	79	66	64	87	73	67
Gender	F	M	F	F	F	M
DAPT	–	+	–	–	–	–
Anticoagulant drugs	–	–	–	+	–	–
Steroid (mg/day)	–	–	Prednisolone (10)	Prednisolone (12.5)	–	Hydrocortisone (20)
Transfusion (units)	0	4	16	0	8	4
Inferior mesenteric artery	Patent	Patent	Patent	Patent	Patent	Patent
Internal iliac arteries	Bilateral patent with calcification	Bilateral patent with calcification	Bilateral patent	Bilateral patent with calcification	Bilateral patent with calcification	One-sided patent
Endoscopic treatment (clipping)	–	+	+	–	+	+
Recurrence	–	+	+	+	–	–

AHRU: acute hemorrhagic rectal ulcer; DAPT: dual antiplatelet therapy

### Patient characteristics

The characteristics of the patients in both groups are shown in **Table 2**. Although the 2 groups were largely similar, the AHRU group had significantly more patients taking oral steroids compared to the non-AHRU group (7% vs. 50%, respectively; *P* < 0.001). In the non-AHRU group, 5 patients were regularly treated with steroids, 2 for post-renal transplant, 2 for rheumatoid arthritis, and 1 for polymyalgia rheumatica. On the other hand, in the AHRU group, 3 patients were taking steroids for adrenal insufficiency, idiopathic thrombocytopenic purpura, or systemic lupus erythematosus.

**Table:** Table 2 Patient characteristics

Variables^a^	Non-AHRU group (n = 74)	AHRU group (n = 6)	P value
Age (years)	72.5 [69,77]	70.0 [66.3,77.5]	0.763
Female sex	25 (34)	4 (67)	0.182
Nonambulatory status before the onset of CLTI	2 (3)	0 (0)	1.000
Nonambulatory status before surgery	28 (38)	4 (67)	0.211
Body mass index (kg/m^2^)	21.2 [19.1,23.5]	22.2 [19.8,23.3]	0.695
Hypertension	58 (78)	5 (83)	1.000
CAD	52 (70)	3 (50)	0.370
Dyslipidemia	37 (50)	4 (67)	0.676
CVD	11 (15)	1 (17)	1.000
Hemodialysis	35 (47)	3 (50)	1.000
Diabetes mellitus	44 (60)	5 (83)	0.399
Smoking	42 (57)	2 (33)	0.401
EF (%)	63 [55,67]	58 [47,63]	0.202
Creatinine (mg/dL)	2.42 [0.73,6.80]	3.48 [1.32,7.29]	0.289
Albumin (g/dL)	3.1 [2.7,3.6]	2.9 [2.6,3.1]	0.210
Hemoglobin (g/dL)	11.0 [9.7,12.6]	10.3 [10.0,11.2]	0.499
GNRI	87.6 [78.1,97.8]	84.0 [77.5,87.9]	0.391
Barthel index	80 [65,90]	90 [83,98]	0.214
Medication			
Aspirin	42 (57)	4 (67)	1.000
Clopidogrel	37 (50)	1 (17)	0.676
Cilostazol	11 (15)	1 (17)	1.000
DAPT	24 (32)	1 (17)	0.731
Anticoagulant drugs	14 (19)	1 (17)	1.000
Statin	35 (47)	3 (50)	1.000
Steroid	5 (7)	3 (50)	<0.001

^a^Data are presented as medians [IQRs] or numbers (%). AHRU: acute hemorrhagic rectal ulcer; CLTI: chronic limb-threatening ischemia; CAD: coronary artery disease; CVD: cerebrovascular disease; EF: ejection fraction; GNRI: geriatric nutritional risk index; DAPT: dual antiplatelet therapy; IQR: interquartile range

### Preoperative limb characteristics

**Table 3** shows the clinical characteristics of the limbs in both groups. There were no significant differences in the Rutherford classification, wound ischemia and foot infection (WIfI) stage, or global limb anatomic staging system (GLASS) stage between the 2 groups.

**Table:** Table 3 Clinical characteristics of the limbs

Variables^a^	Non-AHRU group (n = 74)	AHRU group (n = 6)	P value
Rutherford classification			0.283
4	8 (11)	0 (0)	
5	40 (54)	2 (33)	
6	26 (35)	4 (67)	
WIfI stage			0.401
1	2 (3)	0 (0)	
2	12 (16)	0 (0)	
3	16 (22)	0 (0)	
4	44 (59)	6 (100)	
Wound grade			0.184
0	11 (15)	0 (0)	
1	26 (35)	1 (17)	
2	20 (27)	3 (50)	
3	17 (23)	2 (33)	
GLASS stage			0.646
I	15 (20)	2 (33)	
II	22 (30)	2 (33)	
III	37 (50)	2 (33)	

^a^Data are presented as numbers (%). AHRU: acute hemorrhagic rectal ulcer; WIfI: wound ischemia and foot infection; GLASS: global limb anatomic staging system

### Surgical details and short-term outcomes

**Table 4** summarizes the details of bypass surgery and the short-term results of both groups. There were significantly more patients in the AHRU group who were unable to walk during the postoperative hospital stay (1% vs. 50%, respectively; P = 0.041). The length of hospital stay was significantly longer in the AHRU group (48 vs. 106 days, respectively; P = 0.004). No mortality within 30 days was observed in either group, but hospital death was significantly more likely to occur in the AHRU group (3% vs. 50%, respectively; P = 0.002). There were 2 deaths during hospitalization in the non-AHRU group, due to nonocclusive mesenteric ischemia (NOMI) and aortic stenosis, and 3 deaths in the AHRU group, due to NOMI, acute hemorrhagic gastric ulcer, and sepsis due to infection of the foot.

**Table:** Table 4 Surgical details and short-term outcomes

Variable^a^	Non-AHRU group (n = 74)	AHRU group (n = 6)	P value
Distal anastomosis			0.872
Popliteal artery	22 (30)	1 (17)	
Crural artery	22 (30)	3 (50)	
Infra-malleolar artery	30 (40)	2 (33)	
Operation time ≥406 min (median)	48 (65)	6 (100)	0.077
Blood loss (mL) ≥225 mL (median)	43 (58)	5 (83)	0.225
Postoperative nonambulatory status	1 (1)	3 (50)	0.041
Length of hospital stay (days)	48 [30,83]	106 [91,133]	0.004
30-day mortality	0 (0)	0 (0)	N/A
In-hospital mortality	2 (3)	3 (50)	0.002
Cause of death	NOMI, AS	NOMI, acute hemorrhagic gastric ulcer, Sepsis due to limb infection	

^a^Data are presented as medians [IQRs] or numbers (%). AHRU: acute hemorrhagic rectal ulcer; N/A: not available; NOMI: nonocclusive mesenteric ischemia; AS: aortic stenosis

Regarding the use of albumin as a nutritional index, in the AHRU group, the preoperative serum albumin level was 2.9 g/dL [2.6,3.1], but it decreased to 2.3 g/dL [2.2,2.6] at the onset of AHRU.

### Overall midterm outcomes

The limb salvage rate, survival rate, and AFS rate at 2 years were 89%, 75%, and 67%, respectively.

### Risk factors for AHRU after bypass surgery

According to the univariate analysis, factors associated with the development of AHRU were the use of oral steroids (odds ratio [OR], 13.8; 95% confidence interval [CI], 2.19–86.9; P = 0.005) and postoperative nonambulatory status (OR, 7.22; 95% CI, 1.26–41.4; P = 0.026).

## Discussion

In this study, we analyzed factors associated with AHRU after bypass surgery for CLTI. We demonstrated that the use of oral steroids and postoperative nonambulatory status were related to the development of AHRU. This study is the first to demonstrate an association between CLTI and AHRU.

AHRU is a condition in which irregular or ring-shaped ulcers are formed in the lower rectum, resulting in sudden painless bleeding, and is considered to occur primarily in elderly patients with underlying diseases such as cerebrovascular disease, coronary artery disease, and diabetes mellitus.^5,8,10)^ In 1974, Delancy and Hitch first reported 3 cases of acute, painless, life-threatening rectal ulcers,^1)^ and in 1981, Soeno et al. described similar cases, and this was the first time that the term AHRU was used.^2)^ Moreover, Duff and Wright reported 7 cases with symptoms similar to AHRU.^3)^ The leading theory regarding the etiology of AHRU is that it is caused by impaired blood flow to the mucosa of the lower rectum due to prolonged bed rest.^25)^ Although there have been several reports on AHRU, few articles have shown an association between CLTI patients and AHRU.^1–19)^

Our analysis revealed that taking oral steroids and postoperative nonambulatory status were significant factors. Nakamura et al. reported that positional changes from the lateral to the supine position reduce blood flow in the lower rectal mucosa, which could induce ischemic ulcerative lesions.^25)^ It is reasonable to expect that CLTI patients, who are at high risk of atherosclerosis, would be susceptible to developing AHRU due to prolonged postoperative supine positioning. In the present study, of the 6 patients who developed AHRU, 3 had a postoperative nonambulatory status until discharge from the hospital. CLTI patients with multiple comorbidities often experience difficulty making progress with rehabilitation due to unstable circulation and respiratory conditions after surgery. Even in such cases, it is very important to encourage patients to change their positions frequently from the supine to the lateral position to avoid complete bedriddenness. Moreover, if patients cannot undergo rehabilitation due to lower limb pain, we should focus on pain management by using appropriate painkillers or performing continuous nerve blocks.

The lower rectum is supplied by the middle rectal artery, which branches off the internal iliac artery. These vessels may be affected by atherosclerosis in patients who develop AHRU. In this study, among the patients who developed AHRU, 1 had only one side patent, and 5 had bilateral patent. Of these 5 patients, 4 had calcification of the internal iliac artery. However, in many cases, contrast computed tomography or angiography is not used to confirm blood flow in the internal iliac artery, and the relationship between internal iliac artery lesions and AHRU remains unknown.

Furthermore, steroids interfere with the healing of ulcers, and delayed healing of rectal ulcers can lead to AHRU.^10)^ In the present study, 3 of the 6 patients who experienced rectal ulcers were regularly treated with steroids for adrenal insufficiency, idiopathic thrombocytopenic purpura, or systemic lupus erythematosus. Further studies are needed to investigate the association between steroids and the postoperative onset of rectal ulceration.

Bleeding complications can occur as a complication of antithrombotic drugs. For the patients in this study, continuous heparin injections, antiplatelet agents, and anticoagulant therapy were also used postoperatively. However, there was no difference in antithrombotic therapy between patients with and without AHRU.

Some reports suggest that hypoalbuminemia is a risk factor for AHRU.^11,13)^ In this study, there was no difference in preoperative serum albumin levels between the 2 groups, but in the 6 patients who developed AHRU, serum albumin levels just before the onset of AHRU were lower than the preoperative values. There is a possibility that a decrease in postoperative serum albumin levels compared with preoperative levels may be associated with the development of AHRU; however, the small number of cases in this study makes it impossible to test this hypothesis, and further studies are needed.

It has been reported that AHRU has a high recurrence rate. However, there is not yet a consensus on the risk factors for recurrence.^18)^ In this study, 3 out of 6 patients of AHRU experienced a recurrence. Although active bleeding and circumferential ulceration are considered to be risk factors for recurrence, the number of cases is too small to allow analysis of these risk factors.^18)^ Patients who developed AHRU needed to be managed on a fasting, and this is thought to have led to the extension of their hospital stay.

The limitations of this study should be mentioned. First, this study was performed retrospectively at a single Japanese vascular surgery center. Therefore, our results might not be free from bias. Second, the number of patients was too small. A larger sample size might be necessary to obtain sufficient statistical power to define the relationship between CLTI and AHRU. Finally, no postoperative physical function other than ambulation was measured in our study. These limitations should be considered in future studies, as they could influence the results.

## Conclusions

This study revealed that taking oral steroids and postoperative nonambulatory status were associated with the onset of AHRU after bypass surgery for CLTI. These findings suggest that postoperative rehabilitation is important to prevent the development of AHRU.

## Declarations

### Disclosure statement

The authors have no conflict of interest to disclose.

### Author contributions

- Study conception: YK
- Data collection: YK
- Analysis: YK
- Investigation: YK, MS, HB
- Manuscript preparation: YK, MS, HB
- Critical review and revision: all authors
- Final approval of the article: all authors
- Accountability for all aspects of the work: all authors.
